# Evaluation of [^18^F]tetrafluoroborate as a Potential PET Imaging Agent in a Sodium Iodide Symporter-Transfected Cell Line A549 and Endogenous NIS-Expressing Cell Lines MKN45 and K1

**DOI:** 10.1155/2022/2679260

**Published:** 2022-02-27

**Authors:** Mengda Niu, Jingjing Qin, Liang Wang, Yujia He, Chuanhuizi Tian, Yanyan Chen, Pufeng Huang, Zhiping Peng

**Affiliations:** Department of Radiation Medicine, School of Basic Medicine, Chongqing Medical University, Chongqing, China

## Abstract

[^18^F]tetrafluoroborate (TFB) has been introduced as the ^18^F-labeled PET imaging probe for the human sodium iodide symporter (NIS). Noninvasive NIS imaging using [^18^F]TFB has received much interest in recent years for evaluating various NIS-expressing tumors. Cancers are a global concern with enormous implications; therefore, improving diagnostic methods for accurate detection of cancer is extremely important. Our aim was to investigate the PET imaging capabilities of [^18^F]TFB in NIS-transfected lung cell line A549 and endogenous NIS-expressing tumor cells, such as thyroid cancer K1 and gastric cancer MKN45, and broaden its application in the medical field. Western blot and flow cytometry were used to assess the NIS expression level. Radioactivity counts of [^18^F]TFB, *in vitro*, in the three tumor cells were substantially higher than those in the KI inhibition group in the uptake experiment. *In vivo* PET imaging clearly delineated the three tumors based on the specific accumulation of [^18^F]TFB in a mouse model. *Ex vivo* biodistribution investigation showed high [^18^F]TFB absorption in the tumor location, which was consistent with the PET imaging results. These results support the use of NIS-transfected lung cell line A549 and NIS-expressing tumor cells MKN45 and K1, to investigate probing capabilities of [^18^F]TFB. We also demonstrate, for the first time, the feasibility of [^18^F]TFB in diagnosing stomach cancer. In conclusion, this study illustrates the promising future of [^18^F]TFB for tumor diagnosis and NIS reporter imaging.

## 1. Introduction

The human sodium iodide symporter (NIS) is a plasma membrane protein that mediates the active transport of iodide (I^−^) into cells and is the basis for the clinical utilization of radioiodine [1–3]. Other monovalent anions having ionic radii/-volumes similar to I^−^, such as pertechnetate (TcO_4_^−^), perchlorate (ClO_4_^−^), and tetrafluoroborate (TFB^−^), can also be transported by NIS. And they have been well established as NIS substrates [4, 5]. NIS has a restricted tissue expression, including the thyroid, salivary glands, and stomach, thereby enabling good imaging contrast [5–10]. Importantly, there are no reports on the toxicity of ectopic NIS expression in nonthyroidal cells, and NIS is also unrelated to the host immune response [11–13]. Therefore, NIS is a promising imaging reporter gene for tumor-specific diagnosis and treatment.

NIS-mediated nuclides are primarily iodides, such as [^131^I] and [^123^I] [10]. I^−^ and other iodide analogs such as [^99m^Tc]TcO_4_^−^ are the major tracers for single-photon emission computed tomography (SPECT) and had been the mainstay of clinical molecular imaging [14]. In gene therapy, cell therapy, and cancer metastasis research, molecular imaging methods can explore smaller cell populations in the body. Therefore, the sensitivity and resolution of tracking applications are critical at the cellular level. At the same time, because the imaging can be repeated noninvasively, the number of animals required for the experiment is significantly reduced [15–17]. Although SPECT is widely used, positron emission tomography (PET) is considered to be superior: PET imaging with [^124^I]I^−^ has enhanced sensitivity, resolution, and quantification compared to SPECT imaging with [^131^I]I^−^. Accordingly, PET with [^124^I]I^−^ has gained much interest in recent years for the evaluation of thyroid disease using NIS reporter gene imaging [18, 19]. However, the long half-life, abundant high energy *γ* emissions, low positron yield, and high positron energy of [^124^I]I^−^ can result in a high radiation dose and poor PET imaging quality (compared with other isotopes like [^18^F]F^−^) [20].

As early as the 1950s, Anbar et al. [21] reported the radiolabeling of [^18^F]TFB by isotopic exchange, utilizing [^18^F]F generated by the reactor on nonradioactive TFB under acidic conditions. TFB is a fluorine-containing ion that interacts with NIS. In 2010, Jauregui-Osoro et al. [22] updated the isotope exchange radiolabeling of [^18^F]TFB and reported NIS-specific accumulation of [^18^F]TFB in a preclinical model of normal thyroid tissue and thyroid cancer, proving that TFB is an effective NIS-targeted physiological imaging agent. The potential of [^18^F]TFB as a PET imaging agent for NIS was recently reported on the healthy patients with thyroid cancer [23, 24]. [^18^F]TFB is simple to prepare and has excellent PET imaging properties, including good pharmacokinetics, quick tumor absorption, and fast and almost complete circulatory clearance. Furthermore, PET/computerized tomography (CT) imaging in normal mice revealed that [^18^F]TFB is only absorbed by normal tissues that express NIS, such as the salivary glands, stomach, and thyroid [25–29]. Compared to SPECT and ^124^I-PET, [^18^F]TFB demonstrated superior image resolution, sensitivity, quantification, dosimetry, *in vivo* and *in vitro* stability, and ease of production in PET centers. [^18^F]TFB also showed no undesirable metabolic complications and undue patient radiation burden, which is usually associated with [^124^I]I^−^ [27]. Therefore, we believe that [^18^F]TFB is an effective NIS detector for PET/CT.

Cancer has become a huge global concern, with devastating consequences for human health. As a result, cancer research on diagnosis, treatment, and prognosis has long been a priority [30–32]. [^18^F]TFB has now been utilized to monitor cells or tissues expressing the NIS gene as a promising NIS-PET imaging gene reporter probe, and some studies have reported [^18^F] TFB-PET imaging outcomes of patients with thyroid cancer [15, 23, 24]. Therefore, our research team is dedicated to investigate the potential of [^18^F]TFB as a PET/CT imaging agent for the diagnosis of NIS-expressing cancers to broaden its application [24, 25]. Experimental cancer cell lines expressing reporter genes are useful tools for preclinical and translational research. In this study, the NIS gene was stably transfected into the lung cancer cell line A549, resulting in a cell line (NIS-A549) with high NIS expression levels. Therefore, we used NIS-A549 and two types of tumor cells that endogenously express NIS (thyroid carcinoma cell line K1 and gastric cancer MKN45) to assess the PET imaging capabilities of [^18^F]TFB *in vivo* and to expand its use in fundamental research and clinical treatment.

## 2. Materials and Methods

### 2.1. Cell Culture

The human gastric cancer cell line MKN45, thyroid cancer cell line K1, lung cancer cell line A549, and human embryonic kidney 293T cells are preserved by the Department of Radiological Medicine and Oncology (Chongqing Medical University, Chongqing, China). K1 and 293T cells were cultured in DMEM (Gibco, Thermo Fisher Scientific, USA) supplemented with a 1% penicillin/streptomycin (Beyotime Biotechnology, Shanghai, China) and 10% fetal bovine serum (FBS; Gibco). MKN45 cells and A549 cells were grown in RPMI-1640 (GIBCO) supplemented with a 1% penicillin/streptomycin and 10% FBS. All cells were maintained in flasks at 37°C in a humidified atmosphere containing 5% CO_2_, and the culture medium was replenished when they reached 80% confluency. And all cells in the logarithmic phase of growth were used for all experiments.

### 2.2. Lentivirus Vector Transfection

A NIS overexpression lentiviral vector was constructed by Hanbio Biotechnology (Shanghai, China) and based on pHBLV-CMV-MCS-3FLAG-EF1-ZsGreen-T2A-PURO. NIS overexpression vector was verified by sequencing. The virus packaging system is composed of psPAX2 and pMD2G plasmids. Transfection was performed by using a Lipofilter transfection kit (Hanbio Biotechnology). 293T and A549 cells were transfected with NIS overexpression lentiviral vector, respectively, with a multiplicity of infection of 5, in the presence of polybrene (5 *μ*g/mL, Hanbio Biotechnology) for 24 h. Following transfection, the supernatant, containing the NIS overexpression lentiviral vector, was replaced with fresh RPMI-1640 medium and cultured. After 72 h of puromycin (10 g/ml, Solarbio Life Science) screening, cell lines with stable NIS expression (NIS-A549 and NIS-293T) were produced. The expression of NIS was determined using flow cytometric analysis (FCA) and western blot (WB). The NIS-A549 and NIS-293T cells were also sectioned and subjected to fluorescence microscopy.

### 2.3. Flow Cytometric Analysis

The cells were bound with Human SLC5A5 Alexa Fluor 594-conjugated Antibody (NIS-594-antibody, R&D Systems). A549 and NIS-A549 cells were washed twice with PBS and harvested and then processed into single-cell suspensions containing 5 × 10^6^ cells/mL, which was stained with the abovementioned antibodies in the dark for 30 min at room temperature. The stained cells were centrifuged and washed twice with PBS. Then, the cell samples analyzed by a BD Influx flow cytometer (Beckman CytoFLEX, California, USA).

### 2.4. Fluorescent Staining Assay

The cells were seeded into 6-well plates and incubated at 37°C for 24 h prior to experiments. The cells were fixed in a fixative solution for 10 minutes before being rinsed twice in PBS. The cells were then incubated for 10 min with DAPI staining solution (Beyotime, Shanghai, China) before being rinsed twice more. Green fluorescence protein (GFP) will be expressed in cells that have been successfully transfected. Subsequently, fluorescence signals were detected using a fluorescence microscope (Nikon).

### 2.5. Western Blot Analyses

The cells were harvested, washed three times with PBS, and then lysed by using RIPA buffer supplemented with a 5% 100 mM PSMF. Protein concentrations were determined using BCA Protein Assay Kit (both from Beyotime Biotechnology, Shanghai, China). Equal amounts of total protein (30 *μ*g per lysate) were separated by 10% SDS–PAGE at 250 V for 30 min and then transferred onto PVDF (Millipore Corp, USA) at 350 mA for 50 min. After blocking with 5% nonfat milk powder dissolved in Tris-buffered saline with 0.1% Tween20 (TBST) at room temperature for 60 min, membranes were incubated with rabbit anti-NIS and *β*-actin polyclonal IgG (BIOSS, Beijing, China) overnight at 4°C. The membranes were washed and incubated with a horseradish peroxidase- (HRP-) conjugated goat anti-rabbit IgG (BIOSS, Beijing, China) for 60 min at room temperature. The immunoblotted proteins were visualized with a fluorescence detector (ChemiDoc Touch Imaging System, Bio-Rad, USA) using the enhanced chemiluminescence (ECL) kit (Beyotime Biotechnology, Shanghai, China) at room temperature.

### 2.6. Quantitative Real-Time PCR (qRT-PCR)

For quantification of NIS expression in cells, total RNA was extracted using the Total RNA Quick Purification Kit (BioTeke, Beijing, China) according to the manufacturer's instructions. Reverse transcription was performed using the PrimeScript RT Reagent Kit with gDNA Eraser (Takara, Tokyo, Japan), and qRT-PCR was run with the 2× RealStar Green Power Mixture (GenStar, Beijing, China). Relative expression levels were normalized to the control gene that *β*-actin and mRNA expression was calculated with the 2^-*ΔΔ*Ct^ values. The following sequences were used as primers: NIS: (5′ACACCTTCTGGACCTTCGTG-3′) and (5′-GTCGCAGTCGGTGTAGAACA-3′) and *β*-actin: (5′AGAAAATCTGGCACCACACC-3′) and (5′-TAGCACAGCCTGGATAGCAA-3′).

### 2.7. Synthesis of [^18^F]TFB

[^18^F]TFB was synthesized as previously reported procedure [27]. [^18^F]NaF (HTA Co., Ltd.) in H_2_O was trapped on a quaternary methyl ammonium (QMA) cartridge (Sep-Pak QMA Light, Waters, UK). A volume of 1.0 mL 0.9% NaCl solution (Boster Biological Technology, California, USA) was then passed through the QMA cartridge, eluting most of the [^18^F]F^−^ into a glass vial. The eluate was then dried under a stream of N_2_ at 95°C, followed by repeated azeotropic distillation with acetonitrile (MeCN, Chron Chemicals, Chengdu, China) (3 × 0.5 mL). 15-C-5 (24 mg, Energy Chemical, Shanghai, China) in MeCN (0.5 mL) and BF_3_·OEt_2_ (18.3 *μ*L, Adamas-beta) in MeCN (0.5 mL) were then added into the dried glass vial containing [^18^F]F^−^, and the reaction mixture was heated to 80°C for 30 min. The mixture was passed over a neutral alumina cartridge (Alumina N Plus Lite Sep-Pak, Waters, UK) into a glass vial, and the neutral alumina cartridge was washed with H_2_O (2 mL). This mixture was then passed over a QMA cartridge, and the QMA cartridges were washed with H_2_O (2 mL). The QMA cartridge containing the reaction product was then washed with H_2_O (4 mL). The purified solution was then eluted from the QMA cartridge with 0.9% NaCl (3 mL).

### 2.8. Radioanalytical Methods

Radio thin-layer chromatography (TLC) was performed with a neutral alumina stationary phase (10 × 80 mm; Yantai Jiangyou Silica gel Development, Yantai, China) and methanol (100%) as the mobile phase. The TLC plates were scanned using a radio TLC linear scanner (Eckert & Ziegler) with *β*^+^ probe. The purity of the purified solution with the radioactive product in the reaction solution was determined by the percentage of radioactivity related to the [^18^F]TFB peak in the total detected chromatographic radioactivity.

### 2.9. Cellular Uptake, Inhibition, and Retention Assay

The cells were seeded into 24-well plates at a density of 0.5 × 10^5^ cells/well and incubated for 24 h. Prior to the experiments, each well was washed twice with PBS, and 200 *μ*L medium was added in to each well with or without 100 mM KI. The plates were incubated for 20 min, and 10 *μ*L freshly formulated medium containing [^18^F]TFB (7.4 kBq) was added in to each well. At intervals from 30 to 120 min, the radioactive medium was aspirated and cells were washed twice with PBS. All cells detached with 500 *μ*L of trypsin-EDTA solution (0.05%, Beyotime Biotechnology, Shanghai, China). The uptake radioactivity was counted in a *γ*-counter (Perkin Elmer). For retention experiments, cells were seeded and incubated in 24-well plates with triplicate wells for each time point as described above. The medium was removed, and each well was washed twice with PBS. After addition of fresh 500 *μ*L medium containing [^18^F]TFB (7.4 kBq) to each well and incubation for 2 h, the radioactive supernatant was removed and fresh medium was added to each well. At intervals from 15 to 120 min, the medium was collected and the activity in the cells was extracted with 1 M NaOH (500 *μ*L). Results (counts per minute, cpm) were corrected for decay and subtracted the background measurement.

### 2.10. Subcutaneous Tumor Models

All animals used in this study were performed in accordance with protocols approved by the Guidelines of Chongqing Medical University Biomedical Ethics Committee. Female BALB/c nude mice, 4-6 weeks of age, were purchased from the Vital River Laboratory Animal Technology (Beijing, China) and housed and maintained by the Department of Laboratory Animal Medicine at the Chongqing Medical University. We chose the MKN45, K1, NIS-A549, and A549 cells to establish a tumor model, in which cells stably expressed NIS. We subcutaneously injected 100 *μ*L of MKN45 and K1 cells (5 × 10^6^) into the right armpit of each mouse and injected 100 *μ*L of NIS-A549 and A549 cells (5 × 10^6^) into the right and left armpits of each mouse, respectively. Mice were also injected with the gastric cancer cell line MKN45 and thyroid cancer cell line K1. All mice, experimental and normal, were subjected to subsequent experiments.

### 2.11. PET/CT Imaging

All animal were imaged using a nanoScan® PET/CT (Mediso Medical Imaging Systems, Budapest, Hungary). Based on the preliminary cellular uptake study, 60 min was selected as the best time point because when compared to other time points, a superior imaging result was obtained. Both experimental and normal mice were intravenously injected with 200 *μ*L of [^18^F]TFB (1.85 MBq) and were scanned using PET/CT with static imaging after 60 min postinjection. Mice were anaesthetised using isoflurane (1.5%; RWD Life Science, Shenzhen, China) in oxygen and placed on the scan bed in a prone position. Respiration of mice and bed temperature were monitored during the whole scanning process. Anaesthesia was maintained at 1.5-2% isoflurane throughout the scan. PET image reconstruction and decay correction were performed using the 3D PET image reconstruction software (Mediso, Budapest, Hungary).

### 2.12. Biodistribution

Each tumor-bearing nude mouse was injected with 200 *μ*L [^18^F]TFB (18.5 kBq) through the tail vein. Mice were sacrificed at 30, 60, and 120 min after injection, and tissues (blood, brain, heart, liver, spleen, lung, kidney, stomach, intestine, bone, muscle, and tumor) were collected, blotted dry, and weighed. Radioactivity counts of the tissues and injected drug were tested with a gamma counter. The percentage of injected dose per gram of tissue (%ID/g) was calculated as follows:%ID/g = (tissue radioactivity of the tissues/radioactivity count of injected drug)/tissue mass × 100%.

### 2.13. Statistical Analysis

All quantitative data are displayed as mean ± SD. Normally distributed data were analyzed using the Welch 2-tailed *t*-test (2 groups) or ANOVA with Dunnett post hoc test (3 ≥ groups). Each experiment was independently repeated 3 times. The results were considered significant at a *p* value less than 0.05.

## 3. Results

### 3.1. Functional Verification of NIS Lentivirus

**Figure 1:**
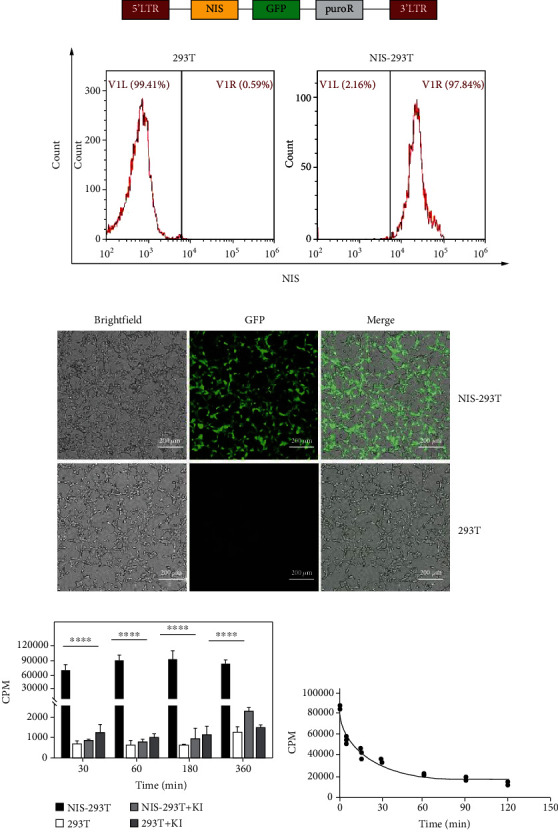
Schematic of the NIS plasmid construct and the functional NIS expression in NIS-293T and 293T cells. (a) Schematic of the NIS plasmid construct. (b) FCA showed the expression of NIS in NIS-293T and 293T cells were 97.84% and 0.59%, respectively. (c) Abundant GFP expression in NIS-293T cells under fluorescence microscopy. (d) ^125^I uptake and inhibition effect of KI in ^125^I uptake in NIS-293T and 293T cells. (e) ^125^I efflux in NIS-293T and 293T cells. ^∗∗∗∗^*p* < 0.0001.

The NIS overexpression lentiviral vector was mainly composed of NIS, GFP, and puromycin resistance genes (Figure 1(a)). After the 293T cells were transfected with NIS lentivirus, FCA showed that 97.84% of the NIS-293T cells expressed NIS (Figure 1(b)), and Fluorescent Assay confirmed the expression of GFP (Figure 1(c)). Therefore, we successfully constructed NIS-293T cells. The rapid accumulation of ^125^I in the NIS-293T cells was observed, but no remarkable accumulation was found in the 293T cells (Figure 1(d)). The addition of KI to the medium considerably prevented the accumulation of ^125^I in the NIS-293T cells, demonstrating that the accumulation was specific and mediated by NIS (Figure 1(d)). After preincubating NIS-293T cells in medium containing ^125^I, replacement of the medium with fresh medium containing no ^125^I led to rapid efflux of ^125^I (Figure 1(e)). Finally, these results showed that the NIS overexpression lentiviral vector prepared in this study was effective.

### 3.2. Expression of NIS Protein in MKN45, K1, NIS-A549, and A549 Cells

**Figure 2:**
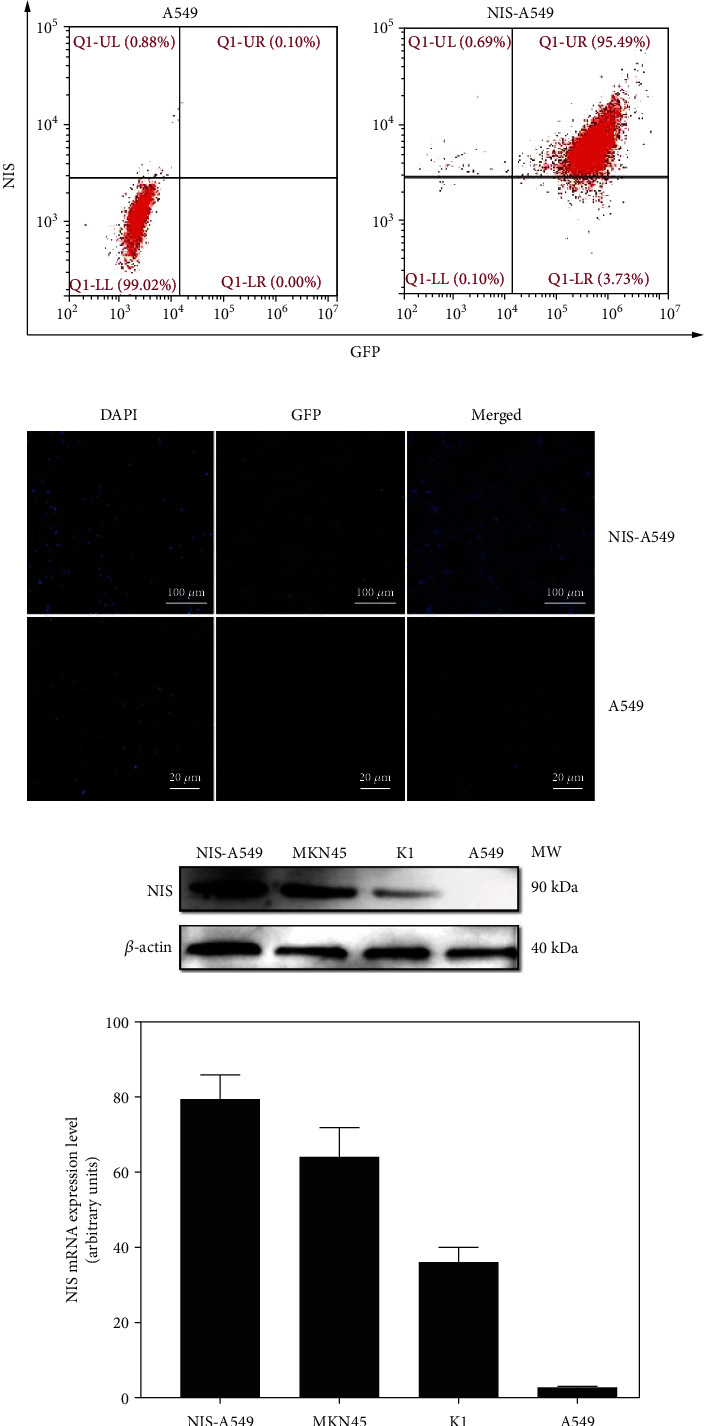
The expression of NIS in NIS-A549, MKN45, K1, and A549 cells. (a) FCA showed the expression of NIS in A549 cells, which was 0.88%, and the expression of NIS and GFP in NIS-A549 cells which was 95.49% and 99.22%, respectively. (b) Abundant GFP expression in NIS-A549 cells under fluorescence microscopy. (c) Western blot showed the expression of NIS in NIS-A549, MKN45, K1, and A549 cells. (d) qRT-PCR revealed high NIS expression in NIS-A549, MKN45, and K1 cells compared to A549 cells (*n* = 3).

FCA verified the presence of NIS and GFP in the NIS-A459 cells (Figure 2(a)), and fluorescence microscopy also verified the presence of GFP in the NIS-A459 cells (Figure 2(b)). More than 95% and 99% of the NIS-A549 cells expressed NIS and GFP, respectively, which proves that we successfully established cell line the overexpresses NIS (Figures 2(a) and 2(b)). WB and qRT-PCR were used to assess the expression of NIS protein in the MKN45, K1, NIS-A549, and A549 cells and confirmed that the comparison of A549, MKN45, K1, and NIS-A549 cells showed higher expression of NIS protein at approximately 90 kDa (Figures 2(c) and 2(d)). The expression of NIS protein in MKN45, K1, and NIS-A549 cells indicated that these cells could be used for subsequent PET/CT scanning in this study.

### 3.3. Radiosynthesis of [^18^F]TFB

**Figure 3:**
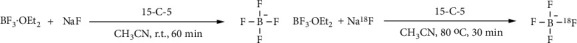
Synthesis of [^18^F]TFB.

**Figure 4:**
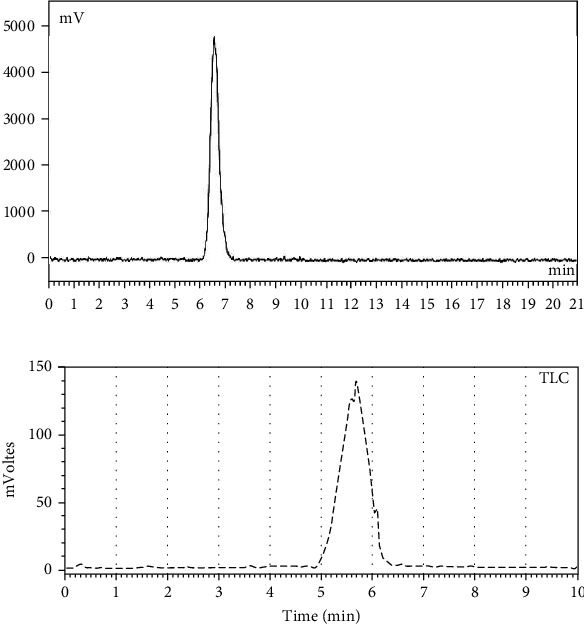
Quality Control of [^18^F]TFB. The activity of the isolated [^18^F]TFB was based on a starting radioactivity of [^18^F]F^−^ with 62.9 MBq. (a) HPLC showing the purified [^18^F]TFB with a retention time (Rt) of 6.59 min. (b) The radio TLC of purified [^18^F]TFB was carried out using a neutral alumina stationary phase with methanol (100%) as the mobile phase. As determined by TLC, the mean *R*_*f*_ value for [^18^F]TFB was about 0.57 on average.

As can be seen in Figure 3, the target compound [^18^F]TFB was successfully synthesized. The crude product was subsequently purified by silica gel chromatography, and the chemical structure was characterized by ^19^F NMR. ^19^F NMR (376 MHz, Chloroform-d) was *δ*-149.89–-149.95. The average radiochemical purity (RCP) of the isolated compound was ≥98% as determined by HPLC (Figure 4(a)), and the TLC data showed that the *R*_*f*_ value of [^18^F]TFB was 0.57 (Figure 4(b)). With a starting radioactivity of ~62.9 MBq, the specific activity of final product was 4.5 ± 2.7 GBq/*μ*moL (*n* = 3). The radiochemical yield was 17.4 ± 1.8% (decay-corrected).

### 3.4. In Vitro Specific Accumulation of [^18^F]TFB in Tumor Cells Expressing the NIS

**Figure 5:**
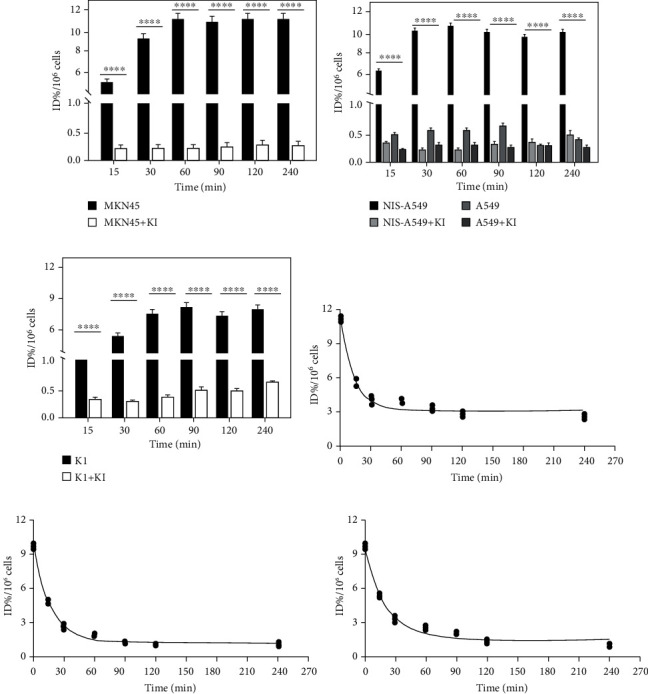
The time course of radiotracer accumulation and efflux of [^18^F]TFB in MKN45, K1, NIS-A549, and A549 cells (*n* = 3). The figure shows the accumulation of [^18^F]TFB in (a) MKN45, (b) NIS-A549 and A549, and (c) K1 using gamma counter, with and without blocking with KI. Data are presented as mean ± 1 SD (%ID/10^6^ cells). Efflux of radioactivity from (d) MKN45, (e) NIS-A549, and (f) K1 cells after treatment with [^18^F]TFB without KI blocking for 120 min, followed by replacement of media with [^18^F]TFB-free media. Error bars represent 1 SD. ^∗∗∗∗^*p* < 0.0001.

Significant uptake of [^18^F]TFB in MKN45 (Figure 5(a)), NIS-A549 (Figure 5(b)), and K1 (Figure 5(c)) was observed and could be blocked by addition of KI. No uptake was observed in the A549 cells (which does not express NIS), either in the presence or absence of KI (Figure 5(b)). The data showed rapid accumulation of [^18^F]TFB in MKN45, NIS-A549, and K1 cells, reached a peak between 30 and 60 min and was maintained for 240 min (Figures 5(a)–5(c)). Thereby, uptake and inhibition experiments demonstrate that the [^18^F]TFB uptake in these cells was specifically mediated by NIS. After preincubating MKN45 (Figure 5(d)), NIS-A549 (Figure 5(e)), and K1 (Figure 5(f)) cells in medium containing [^18^F]TFB, replacement of the medium with fresh medium containing no [^18^F]TFB led to rapid efflux of [^18^F]TFB more than 65.07 ± 0.23%, 79.64 ± 0.40%, and 74.33 ± 1.56% within 60 min, respectively, followed by a slow decrease in the retention rate. This indicates that [^18^F]TFB can be quickly removed from cells within 60 min in a nonradioactive environment, thus reducing the impact on cells.

### 3.5. PET/CT Imaging

**Figure 6:**
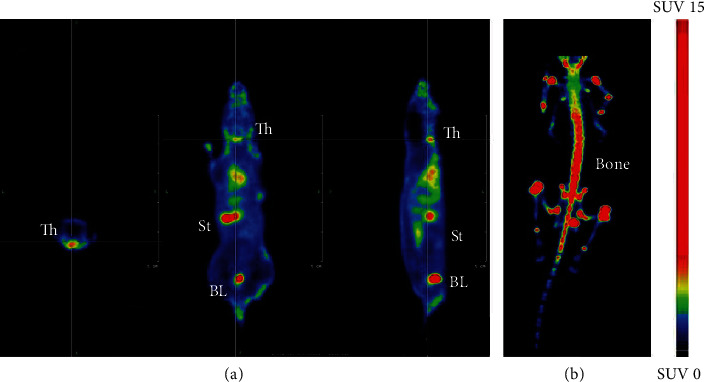
Static scans of [^18^F]TFB in normal mice. PET imaging of (a) a normal BALB/c mouse at 60 min postinjection of [^18^F]TFB (3.7 MBq) showing uptake in the thyroid (Th), stomach (St), salivary gland kidney, and bladder (BL). Representative images of (b) normal Kunming mouse at 60 min postinjection of [^18^F]NaF (14.8 MBq) showing uptake almost in the bone.

**Figure 7:**
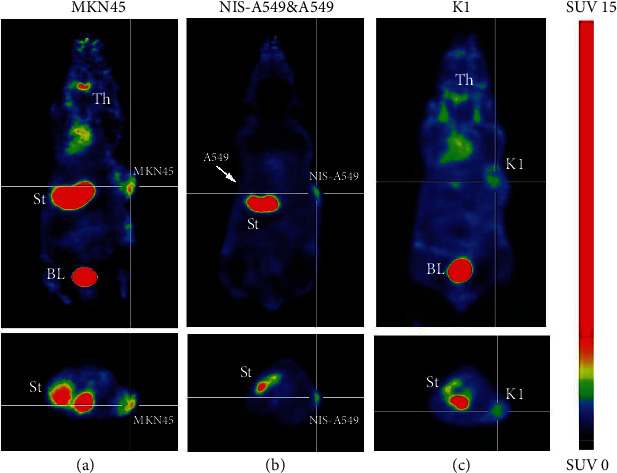
Static scans of [^18^F]TFB in mice bearing MKN45, K1, A549, and NIS-A549 cells at 1 h PET images of [^18^F]TFB distribution at at 1 h i.v. in mice bearing (a) MKN45, (b) A549 and NIS-A549 cells, and (c) K1 showing uptake in the thyroid (Th), stomach (St), bladder (BL), salivary gland, and tumor tissue. White cross targets designate the location of NIS^+^ tumors. White arrows designate location of NIS^−^ tumor without detectable uptake of [^18^F]TFB.

Whole-body static PET-CT imaging of [^18^F]TFB was studied at 60 min post-i.v. in BALB/c nude mice bearing both MKN45, K1, A549 and NIS-A549 xenografts, and normal mice. The [^18^F]TFB-PET imaging data from normal nude mice demonstrated a normal distribution (thyroid, salivary glands, stomach, kidney, and bladder) of [^18^F]TFB (Figure 6(a)). In addition, the [^18^F]NaF-PET image data of ordinary Kunming mice (Figure 6(b)) revealed that the major signals were nearly entirely focused on the bone, demonstrated the successful synthesis of [^18^F]TFB. As expected, PET imaging data (60 min postinjection) in BALB/c nude mice bearing both MKN45 (Figure 7(a)), K1 (Figure 7(c)), and A549 and NIS-A549 (Figure 7(b)) xenografts revealed remarkable uptake of the radiotracer in the tumors and organs (thyroid, salivary glands, and stomach) endogenously expressing NIS. [^18^F]TFB was excreted via the renal route, as demonstrated by radiotracer amounts residing in kidneys and bladder at the imaging time point. We also observed the uptake of the radiotracer by the thyroid and salivary glands, but because those organs were not the focus of our research, we did not analyze them further. The PET images demonstrated NIS specificity of [^18^F]TFB uptake in vivo by the following observations: (i) [^18^F]TFB showed robust uptake in NIS-positive tumors, but no significant tracer uptake was observed in NIS-negative tumors (Figure 7(b)); (ii) robust uptake of [^18^F]TFB was also observed in the thyroid, stomach, kidney, bladder, and NIS-positive tumors of the MKN45 and K1 xenograft mouse models, respectively (Figures 7(a) and 7(c)); (iii) there was no significant uptake in the bones, corroborating the results of negligible defluorination of [^18^F]TFB in mice. Collectively, these results demonstrated the superior stability and good tumor-targeting properties of [^18^F]TFB.

### 3.6. Biodistribution

**Figure 8:**
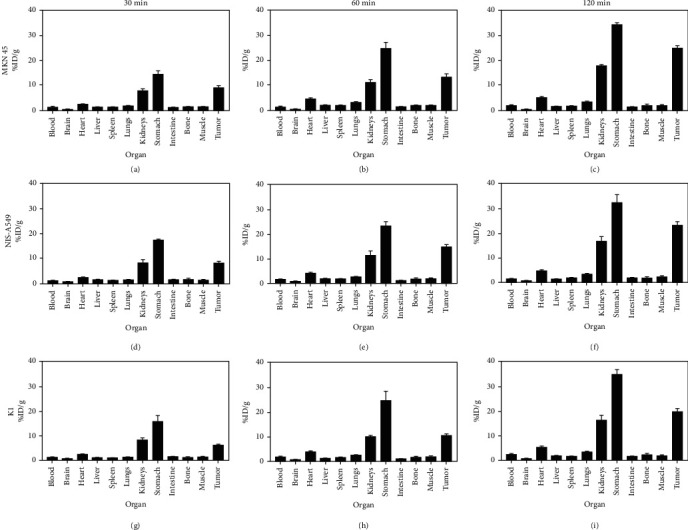
Quantitative analysis of ex vivo biodistribution data. (a–i) *Ex vivo* biodistribution data for [^18^F]TFB in mice bearing MKN45, NIS-A549, and K1 cells at 30, 60, and 120 min postinjection, respectively, determined by counting of tissues (*n* = 3). Relative uptake in organs is represented as mean ± SD (ID%/g). Error bars represent 1 SD.

The biodistribution data are shown in Figure 8; similar to the results of PET imaging, they showed high radioactivity accumulation of [^18^F]TFB in the tumor tissues, heart, stomach, and kidneys. The stomach showed the highest radioactivity uptake because it endogenously expresses NIS. The high radioactivity in kidneys suggested that they are the major organs involved in radioactive marker metabolism, that is, [^18^F]TFB is mainly excreted through the urinary system. Over time, the uptake of [^18^F]TFB by tumors, stomach, and kidneys gradually increased. At 120 min, the uptake of [^18^F]TFB by tumor tissues was 25.57 ± 0.68%ID/g, 22.94 ± 1.35%ID/g, and 19.51 ± 1.33%ID/g (*n* = 3) in MKN45 (Figure 8(c)), NIS-A549 (Figure 8(f)), and K1 (Figure 8(i)) tumor-bearing nude mice, respectively. These values were 14.72, 9.41, and 8.59 times higher than in the muscle uptake and 12.88, 19.56, and 8.29 times higher than in the blood uptake. Notably, the low uptake in the bone implies that there was no defluorination *in vivo*, indicating that [^18^F]TFB was stable in the body. No significant uptake of radioactive markers was observed in the brain tissue, which indicated that [^18^F]TFB could not cross the blood-brain barrier.

## 4. Discussion

Molecular imaging has been used to successfully diagnose diseases such as cancer, inflammation, and neurological and cardiovascular disease and to assess drug resistance, tumor progression, and disease prognosis [33–36]. Recent research has focused on the use of a reporter gene in molecular imaging, which involves transfecting cells with a PET reporter gene that encodes a protein that is selectively targeted by a radiolabeled reporter probe [13, 36]. The application of noninvasive imaging methods, such as PET, and reporter gene imaging systems has demonstrated the capacity to visualize tumor retention/death, trafficking/targeting, and proliferation/expansion *in vivo*. Information on the distribution of target tissues will enable more precise and effective therapy to fight against off-target effects *in vivo*, which may be accomplished by using an appropriate reporter gene-probe imaging system, such as NIS-[^18^F]TFB imaging system [36–38]. The ideal reporter gene-probe imaging system has several characteristics, including (i) the lack of immunogenicity, (ii) an exclusive specificity for target tissues, (iii) a readily available imaging agent, (iv) a feasible imaging method, and (v) the ability to produce a high signal-to-noise ratios using widely available clinical imaging equipment. These reporter systems primarily include the NIS-[^124^I]I^−^ and -[^18^F]TFB, the human norepinephrine transporter (hNET)/[^123^I/^124^I]I^—^MIBG, and [^18^F]MFBG, among others [36]. We chose NIS-[^18^F]TFB as a reporter gene imaging system to assess NIS-expression tumor cells because it satisfies the majority of the aforementioned criteria [28].

Following the cloning of the NIS gene, it has been widely studied as a reporter gene in gene therapy and molecular imaging throughout the last two decades. NIS, a human gene, is now a symporter for imaging agents used in a range of modalities such as PET and SPECT imaging. In the field of molecular reporter gene imaging system research, good imaging tracers are essential for obtaining effective imaging results along with advanced imaging equipment. Therefore, researchers are becoming increasingly interested in and focused on developing new molecular imaging tracers or improving the current tracers. In general, a qualified imaging tracer should have the following properties (or portions of them): high specificity and affinity in the target tissues, structural stability, rapid clearance *in vivo*, low accumulation in nontarget tissues, and should be biochemically safe.

Here, we evaluate a recently introduced PET tracer, [^18^F]TFB, *in vivo*. [^18^F]TFB was successfully synthesized with high radiochemical and chemical purity. The specific activity of [^18^F]TFB (4.5 GBq/*μ*mol) found in this study is slightly lower than that reported previously [27], but it still avoids the saturation of [^18^F]TFB uptake by NIS-expressing tissues, since the instantaneous *in vivo* extracellular BF_4_^−^ concentration can be kept below 0.1 *μ*M. The high specific activity minimizes the pharmacological dose administered, allowing the radioactivity dose to be optimal.

We used NIS-transfected lung cell line A549 and gastric cancer MKN45 and thyroid cancer K1 tumor cells that endogenously express NIS. These cell lines have proved to be a convenient cell models for the investigation of novel NIS substrates and the accumulation kinetics of radiotracers that are substrates for NIS. *In vitro* experiments showed that these cells have an obvious uptake of [^18^F]TFB. Moreover, this accumulation was inhibited by KI, a known specific inhibitor of NIS [29], demonstrating that the accumulation of [^18^F]TFB in these tumor cells is specifically mediated by NIS. As the parent cell lines, MKN45, K1, and A549, can form human xenografts in nude mouse, these cells may be suitable for assessing NIS targeting of tracers in tumor models *in vivo* using PET or SPECT imaging. The uptake of organ-specific tracer by NIS in the stomach and thyroid would limit its application for imaging tumor cells in these anatomical regions. One possible alternative is to orally consume radiopaque chemicals before imaging to block absorption in the stomach. This may be accomplished by using clinically accessible reagents such as barium sulfate. [^18^F]TFB can detect primary tumors expressing NIS *in vivo*; however, both *in situ* gastric cancer cells and normal gastric cells express NIS and, therefore, cannot be correctly differentiated by PET imaging gastric cancer tumors spread easily and have a high proclivity to infiltrate and metastasize. Therefore, anatomical imaging approaches, such as CT and gastroscopy, are ineffective for reliable diagnosis. As a result, we primarily used the NIS-[^18^F]TFB-PET imaging system to detect stomach cancer metastases, complement to clinical diagnostic procedure. Our PET images showed a high uptake in the chest, the cause of which is still unknown. There is research indicating that it may be the trachea, which we will investigate further later. Furthermore, NIS-A549 has a weak PET imaging impact. We believe this may be due to the small size of tumor, which absorbs less [^18^F]TFB, resulting in poor contrast with the background. K1 had good imaging results, indicating that [^18^F]TFB may replace ^131^I in the diagnosis of thyroid cancer. As is widely known, safety concerns must be taken carefully in future clinical translation. A significant disadvantage of using ^131^I is that it is a high-energy radionuclide with a long half-life, and it may remain in the patient's body for a considerable period of time, increasing the risk of normal organs exposed to radiation. Hence, it is easy to conceive that ^131^I may be harmful to patients. Safety concerns must be taken carefully in future clinical translation. Therefore, low-energy radionuclides with short half-lives, such as ^111^In, ^18^F, or ^124^I, are now being chosen for improved nano-PET/CT or nano-SPECT/CT imaging. [^18^F]TFB can lessen the radiation burden on patients because of the short half-life of ^18^F. Our collective imaging findings of [^18^F]KF (nearly all bone uptake) in normal Kunming mice and [^18^F]TFB in normal BALB/c nude mice demonstrated the successful synthesis and stability of [^18^F]TFB.

To the best our knowledge, this is the first study to report that NIS-transfected A549 and tumor cells endogenously expressing NIS are an convenient cell model for the investigation of [^18^F]TFB as a tracer. Moreover, this study confirmed for the first time the feasibility of using [^18^F]TFB in diagnosing gastric cancer *in vivo*. Research of Khoshnevisan et al. [27] has focused on describing a new synthetic approach that can provide an alternative for production of [^18^F]TFB, with higher specific activity and sufficient yield and without the need for a high starting activity of [^18^F]F, thereby removing the risk of NIS saturation *in vivo* even in mice. Our main goal was to evaluate the imaging potential of [^18^F]TFB as a PET imaging agent in the NIS-expressing cell or tumor model and broaden its application in the medical field. In a subsequent study, we will conduct a dynamic scan of PET to explore the metabolic process of the [^18^F]TFB in mice so that the tracer can be used effectively and more comprehensively.

This study established that the NIS-[^18^F]TFB imaging system demonstrated good imaging effects, such as high specificity, sensitivity, and stability, in tumor models. Thus, we believe that the NIS-[^18^F]TFB imaging system has promising prospects for the diagnosis and treatment of tumor using nuclear medicine. Importantly, NIS-[^18^F]TFB established in this study also has different applications: for example, molecular imaging is currently emerging as promising diagnostic approach not only for cancer treatment but also for transplant immunology and regenerative medicine [28]. Whole-body *in vivo* imaging-afforded cell monitoring applications are also becoming more critical for effective development and translation, especially in the context of therapeutic safety.

## 5. Conclusion

[^18^F]TFB was easily synthesized in this study and shown promising capabilities as a PET probe for tumor imaging. Both *in vitro* and *in vivo* data indicated that [^18^F]TFB had strong tumor-targeting properties toward MKN45, NIS-A549, and K1 cells. In summary, our findings suggest that [^18^F]TFB has the potential to be developed as a tumor probe for clinical NIS-PET imaging and to increase the role of NIS in nuclear medicine in the future.

## Data Availability

The data used to support the findings of this study are included within the article.
